# Use of microRNAs in directing therapy and evaluating treatment response in colorectal cancer

**DOI:** 10.1590/S1679-45082014MD2839

**Published:** 2014

**Authors:** Silmara Cristiane da Silveira Andreoli, Nina Jardim Gasparini, Gisele Pereira de Carvalho, Bernardo Garicochea, Robert Edward Pogue, Rosângela Vieira de Andrade

**Affiliations:** 1 Universidade Católica de Brasília, Brasilia, DF, Brazil; 2 Pontifícia Universidade Católica do Rio Grande do Sul, Porto Alegre, RS, Brazil; 3 Centro de Oncologia Sírio Libanês, São Paulo, SP, Brazil

**Keywords:** MicroRNAs, Biological markers, Colorectal neoplasms/chemotherapy, Treatment outcome, Combined modality therapy

## Abstract

Colorectal cancer is the third most common cancer worldwide. Survival and prognosis depend on tumor stage upon diagnosis, and in more than 50% of cases, the tumor has already invaded adjacent tissues or metastasis has occurred. Aiming to improve diagnosis, clinical prognosis and treatment of patients with colorectal cancer, several studies have investigated microRNAs as molecular markers of the disease due to their potential regulatory functions on tumor suppressor genes and oncogenes. This review aimed to summarize the main topics related to the use of microRNAs in diagnosis, clinical prognosis and evaluating treatment response in colorectal cancer.

## RESUMO

O câncer colorretal é o terceiro tipo de câncer mais comum em todo o mundo. A sobrevivência e o prognóstico dependem do estágio do tumor no diagnóstico, momento em que, em mais de 50% dos casos, o tumor já invadiu tecidos adjacentes ou ocorreu metástase. Objetivando-se melhorar o diagnóstico, o prognóstico clínico e o tratamento de pacientes com câncer colorretal, vários estudos investigaram microRNAs como marcadores moleculares da doença, devido à sua função reguladora potencial sobre genes supressores de tumor e oncogenes. Esta revisão procura resumir os principais tópicos relacionados ao uso de microRNAs no diagnóstico, na determinação do prognóstico clínico e na avaliação de resposta ao tratamento do câncer colorretal.

## COLORECTAL CANCER

The role of epigenetics in colorectal cancer (CRC) carcinogenesis is opening an exciting new field of research for translational medicine. Among the many molecules that can interfere in gene expression, long-strand and short-strand RNAs are directly involved with different levels of DNA-protein regulation that seems to be of extreme importance in colorectal carcinogenesis. Among the short-stranded RNAs involved in epigenetics, the microRNAs (miRNA) are among the most often studied so far. Their role in cancer development was initially suggest by Calin et al.^(1)^. Subsequent studies reinforced this new paradigm challenging the previous “carcinogenesis multistep mutation cascade” proposed by Faeron and Vogelstein,^(2)^ while at the same time introducing new potential targets for diagnosis, prevention, and treatment.

### MicroRNAs

MiRNAs arise as a precursor of approximately 70 nucleotides with a characteristic structure, called the primary transcript (pri-miRNA) transcribed by RNA polymerase II. Pri-miRNA is processed by a complex consisting of Drosha and Pasha (also known as DGCR8) proteins located in the cell nucleus. The result of this processing, the precursor miRNA (pre-miRNA), is exported from the nucleus to the cytoplasm and undergoes an enzymatic process catalyzed by the Dicer enzyme, associated with a double strand RNA-binding domain called Loqs-TRBP. This process will lead to the removal of the hairpin loop, resulting in a double-stranded miRNA, which will integrate into the RNA-induced silencing complex (RISC).^(3)^

**Figure 1:**
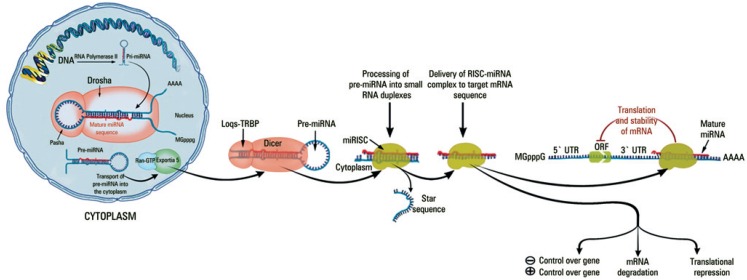
Mechanisms of microRNA maturation

Source: Slack FJ, Weidhaas JB. MicroRNA in cancer prognosis. N Engl J Med. 2008;359(25):2720-2.

The RISC is composed of a set of proteins, of which the most important is Argonaute (Ago). The composition of proteins and the number of copies of Ago depend on the complex and species.^(3)^ The double-stranded product of Dicer processing enters the RISC pathway, resulting in the elimination of one of the strands, leaving the mature single-stranded miRNA associated with the effector protein Ago, as shown in figure 1. While base-matching between the miRNA and the mRNA is more extensive in plants, a core sequence consisting of nucleotides 2-8 seems to determine the consequences in animals, with a perfect match guiding the mRNA towards degradation, and a less than 100% match causing an inhibition of translation.^(4)^ Cleavage is mediated through the Ago protein, however the details of the process remain to be elucidated. Furthermore, it is not yet known whether the transcriptional repression mediated by an imperfect match occurs at translation initiation or post-initiation, with studies pointing to both.^(5)^

## CHEMO-RADIOTHERAPY WITH CAPECITABINE

One of the few evaluations of miRNA expression in humans as a response to chemotherapy was carried out in relation to chemo-radiotherapy with capecitabine (a precursor of 5-fluorouracil) in patients with rectal adenocarcinoma in stages II-III, according to cancer staging methodology of the Union for International Cancer Control (UICC), without distant metastases. Nine miRNAs (miR-145, miR-339, miR-212, miR-125b, miR-137, miR-10a, miR-361, miR-213, and miR-31) were evaluated after 2 weeks of therapy.^(6)^

Only miR-125b and miR-137 demonstrated a consistent expression pattern in most tumors, showing a significant induction for most patients after chemotherapy. This study related increased levels of these miRNAs to a poor therapeutic outcome, since it is believed that miR-125b is involved in processes of cell proliferation and maintenance, and miR-137 is slightly overexpressed in more advanced stages of CRC.^(6)^

## OXALIPLATIN

A study correlating clinical response to doublets, including oxaliplatin as first-line treatment for metastatic CRC with miRNA expression, identified high expression of miR-625-3p, miR-181b, and miR-27b to be associated with poor clinical response.^(7)^ There is a lack of clinical studies with irinotecan and miRNA expression, but one small study^(8)^ that examined the functional significance of the miR-18a *in silico* search showed DNA breaks (a mechanism of cell death induced by irinotecan) by directly suppressing ATM, a key enzyme in DNA damage repair.

## RESVERATROL

A drug that is at the stage of pre-clinical trials for cancer prevention, which is called resveratrol (trans-3.40.5-trihydroxystilbene), was associated to alterations in the pattern of miRNA expression.

The study observed the effect of resveratrol in human SW480 colon cancer lines and it demonstrated that treatment with this drug decreases the levels of oncogenic miRNAs-targeting genes that encode key effectors of the transforming growth factor beta (TGF-β) signaling pathway and tumor-suppressor factors such as PTEN and PDCD4, as well as Dicer-1, which is responsible for catalyzing the enzymatic process that changes pre-miRNA into mature mi-RNA.^(9)^

Resveratrol is also capable of increasing the levels of miR-663, a tumor-suppressor miRNA that targets TGF-β1 transcripts. The results of this study indicate that since, it is possible to manipulate the levels of important miRNAs, like miR-663. Resveratrol could have its anti-metastatic and anticancer effects increased.^(9)^

## CONCLUSION

Accumulated data on the levels of microRNA expression in tumors and their high degree of involvement in the development and progression of cancer identify these molecules as promising candidates to direct cancer therapy and also to predict chemotherapy outcomes. This is possible due to the activities of microRNAs in relation to expression of proteins encoded by oncogenes. The large number of microRNAs in the human genome and the abundance of mRNAs controlled by these molecules suggest a wide range of possibilities offered by the expression patterns of microRNAs.

This analysis of microRNA could be a promising tool for determining the sensitivity of tumors to chemotherapy, as well as which patients would need a more aggressive agent at the beginning of the treatment. However, most of the available data on changes in microRNA expression profiles are limited since they regard information from cell lines of colorectal cancer. Thus, one may highlight the importance of further *in vivo* studies with a larger number of patients, to establish the microRNA expression patterns required to accurately predict the best treatment strategy, making microRNA biomarkers of great importance in clinical practice.
